# Effect and mechanism of GPR75 in metabolic dysfunction-related steatosis liver disease

**DOI:** 10.7150/ijms.101094

**Published:** 2024-09-09

**Authors:** Shuo Wang, Shan Gao, Fei Wang

**Affiliations:** 1 Department of Internal Medicine, The Affiliated Zhong Shan Hospital of Dalian University, Dalian, 116001, China.; 2 Department of Central laboratory, Central Hospital of Dalian University of Technology, Dalian, 116033, China.; 3 Department of Gastroenterology, Affiliated Zhongshan Hospital of Dalian University, Dalian, 116001, China.; 4 Gastrointestinal Endoscopy, Affiliated Zhongshan Hospital of Dalian University, Dalian, 116001, China.

**Keywords:** Metabolic dysfunction-related steatosis liver disease, G-protein coupled receptor 75, Insulin resistance.

## Abstract

Research on G protein-coupled receptor 75 (GPR75) in metabolic dysfunction-related steatosis liver disease (MASLD) reveals its potential role in regulating body weight and energy balance. Loss-of-function mutations in the GPR75 gene are significantly associated with lower body mass index and reduced body weight. Studies demonstrate that GPR75 knockout mice exhibit lower fasting blood glucose levels, improved glucose homeostasis, and significant prevention of high-fat diet-induced MASLD. The absence of GPR75 reduces fat accumulation by beneficially altering energy balance rather than restricting adipose tissue expansion. Moreover, female GPR75 knockout mice show greater protection against lipid accumulation on a high-fat diet compared to males, potentially attributed to higher physical activity and energy expenditure. However, current research primarily relies on mouse models, and its applicability to humans requires further validation. Future studies should explore the role of GPR75 across diverse populations, its clinical potential, and delve into its specific mechanisms and interactions with other metabolic pathways. Ultimately, targeted therapies based on GPR75 could offer novel strategies for the prevention and treatment of MASLD and other metabolic disorders.

## Introduction

Non-alcoholic fatty liver disease (NAFLD) is one of the most common chronic liver diseases worldwide. Its spectrum includes non-alcoholic fatty liver, non-alcoholic steatohepatitis (NASH), cirrhosis, and hepatocellular carcinoma 1. The Delphi Consensus recommends replacing NAFLD and metabolic dysfunction-related fatty liver disease (MAFLD) with a new term called metabolic dysfunction-related steatosis liver disease (MASLD), which not only changes the nomenclature but also modify the definition 2. MASLD is used as an umbrella term through a broader range of metabolic risk factors and can co-exist with other chronic liver diseases. Current epidemiological surveys indicate that MASLD has become the most common chronic liver disease globally and the primary cause of elevated serum transaminase levels in health check-ups 3. It has surpassed viral hepatitis to become the leading chronic liver disease in China 4. Overweight and obesity frequently coexist with MASLD, affecting about 75% of overweight individuals and 90% of those with extreme obesity 5. Obesity also significantly increases the mortality rate in MASLD patients. Obesity-associated MASLD is thought to be associated with unhealthy and dysfunctional adipose tissue that does not expand efficiently to store excess fat, leading to the accumulation of lipids in ectopic sites such as the liver 6. Metabolic syndrome and insulin resistance are major risk factors for MASLD 7. Patients with MASLD and NASH may experience complications that further increase their risk. Therefore, extensive research is urgently needed to uncover the pathophysiological mechanisms of MASLD and identify effective therapeutic targets to mitigate these threats.

**Figure 1:**
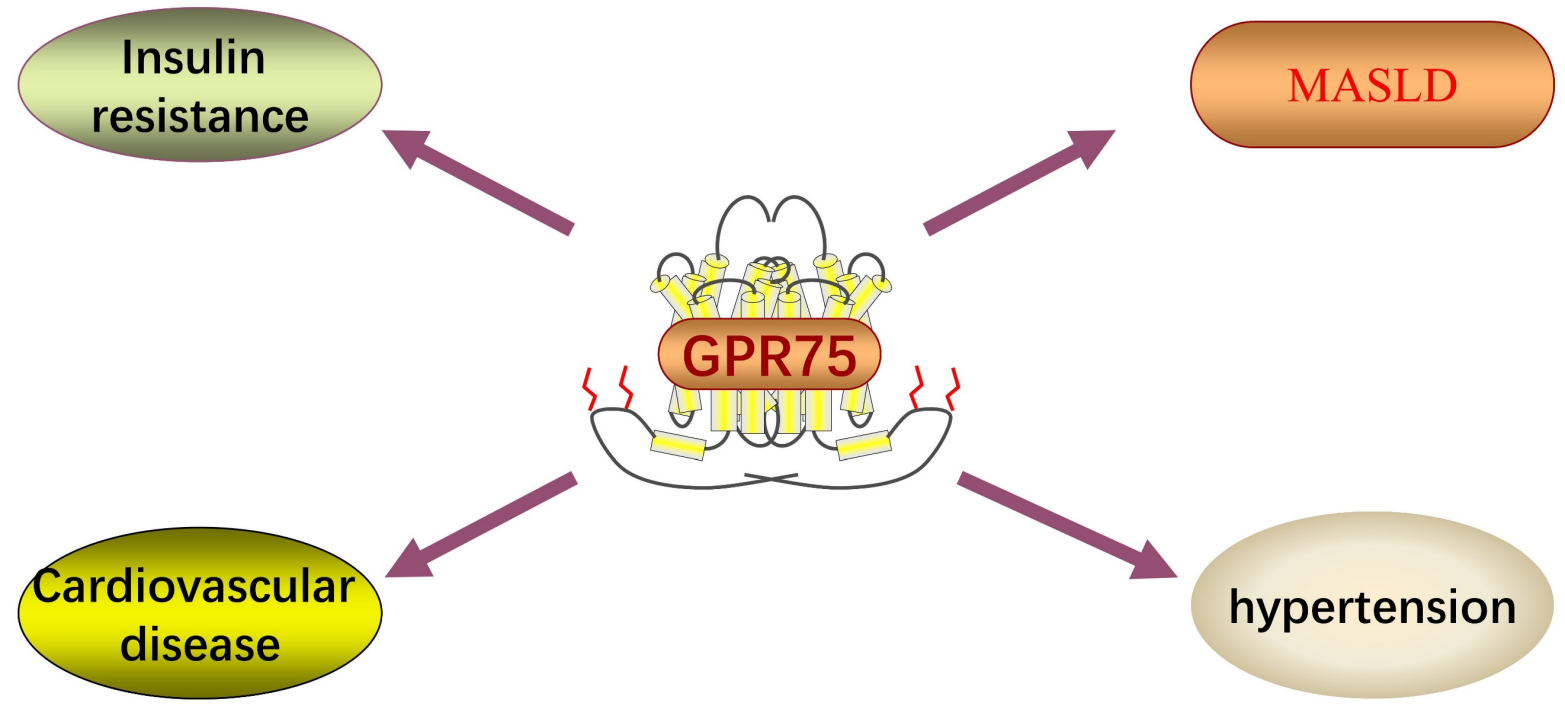
GPR75 has been linked to a variety of diseases in humans.

G-protein coupled receptors (GPCRs) are important membrane molecules involved in regulating physiological responses to hormones, neurotransmitters, and various environmental stimuli. It is estimated that up to 40% of approved clinical drugs exert their effects by targeting GPCRs 8. G protein-coupled receptor 75 (GPR75), a member of the GPCR family, was discovered about 20 years ago, yet its functions are still not fully understood, and debates continue regarding its potential endogenous agonists/antagonists. Current research on GPR75 is limited, but existing studies suggest its significant roles in the liver and cardiovascular system (Figure 1). Here, we will briefly discuss the GPR75 signaling pathway and its mechanism of action in MASLD.

## Structure and Signaling Pathways of GPR75

GPR75 was first reported by Tarttelin et al. in 1999. It is a protein composed of 540 amino acids located on human chromosome 2p16, containing two exons. The first exon includes an untranslated sequence, while the second exon contains the complete coding region of GPR75, with no similarity to other known genes or transcripts 9. GPR75 has typical structural features of GPCRs, including seven transmembrane domains, N-terminal glycosylation sites, and a C-terminal with multiple serine and threonine phosphorylation sites, but it has low structural homology with other similar receptors 10. GPR75 is widely expressed in the liver, vascular endothelium, eyes, kidneys, and adipose tissue, playing an important role in the signaling mechanisms of metabolic syndrome 11.

20-hydroxyeicosatetraenoic acid (20-HETE) is a bioactive lipid produced by cytochrome P450 hydroxylation of arachidonic acid, playing a crucial role in the progression of insulin resistance, obesity, and metabolic syndrome 12, 13. GPR75 has been identified as a selective receptor for 20-HETE, interacting with high affinity 11. Molecular modeling studies suggest that the binding site for 20-HETE might be located in the fifth and sixth transmembrane domains of GPR75 rather than the N-terminal cavity formed by the transmembrane loops. Key residues involved in the interaction between 20-HETE and GPR75 include serine 205, threonine 212, and serine 219^14^. Activation of GPR75 and its ligand 20-HETE triggers pro-inflammatory and hypertensive signaling pathways, leading to diabetes, obesity, endothelial dysfunction, cell proliferation, hypertension, and cardiovascular diseases 15. However, the signaling pathways of GPR75 are not thoroughly studied, and more research is needed to understand the specific molecular mechanisms.

**Figure 2:**
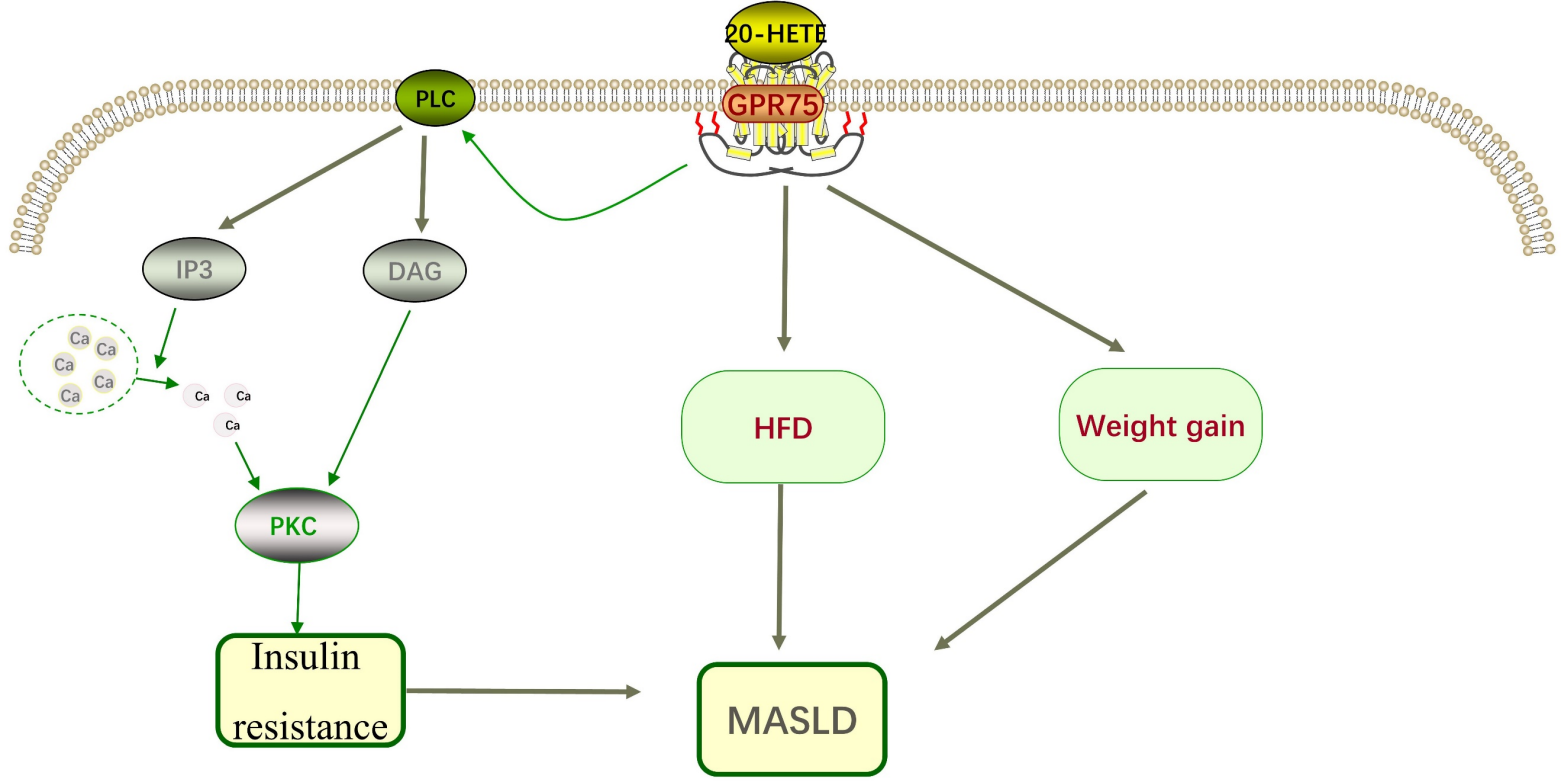
Mechanism of GPR75 in Metabolic Dysfunction-related Steatosis Liver Disease.

Animal studies have shown that obesity and insulin resistance are related to 20-HETE 16. In patients with metabolic syndrome, 20-HETE is associated with elevated triglycerides and reduced high-density lipoprotein 17. To assess the role of GPR75 in 20-HETE-induced EGFR phosphorylation, specific siRNA targeting GPR75 was used, revealing that the effects of 20-HETE were completely inhibited in this context 18, 19. The interaction between 20-HETE and GPR75 can stimulate the IP3/DAG signaling pathway, increasing intracellular calcium levels and activating Protein kinase C-α (PKCα). PKCα then activates phosphatases responsible for dephosphorylating insulin receptors, preventing their activation 20, contributing to insulin resistance, which plays a significant role in the pathogenesis of MASLD and represents a potential therapeutic target (Figure 2). Current research has confirmed that targeting insulin resistance can improve the pathological state of MASLD.

GPR75 also plays a role in cardiovascular pathways. The binding of 20-HETE to GPR75 can trigger signaling pathways in vascular endothelial and smooth muscle cells, leading to vascular angiotensin-converting enzyme expression, hypertension, endothelial dysfunction, remodeling, and contraction 11. The interaction of 20-HETE and GPR75 results in the dissociation of Gαq/11 from GPR75 and associated GPCRK-interacting protein 1 (G1T1), ultimately leading to the phosphorylation of the calcium-activated potassium subunit β of the large conductance voltage and calcium-activated K^+^ channel, resulting in its inactivation and vascular contraction 15. GPR75 as a target for cardiovascular disease was proposed by Garcia et al. 11. In the search for potential vascular system 20-HETE receptors, they observed GPR75 expression on vascular endothelial cells, initiating a series of events leading to vascular contraction upon activation. Additionally, 20-HETE is believed to induce angiotensin-converting enzyme synthesis through a GPR75-dependent mechanism 11. Notably, GPR75 knockout in animal models can inhibit 20-HETE-mediated hypertension, vascular dysfunction, and remodeling. Renal microvascular remodeling is often associated with chronic hypertension, which has been shown to be dependent on 20-HETE.

Ignatov et al. demonstrated that GPR75 activation is achieved by regulating Regulated upon Activation, Normal T-cell Expressed and Secreted (RANTES), a protein consisting of 68 amino acids, known as CeC chemokine ligand 5 (CCL5) 10. Studies have shown that CCL5 exerts insulin secretion effects through GPR75^21^. CCL5 is expressed only in the α-cells of mouse islets, whereas both α and β cells in human islets express CCL5^21^. It was observed that CCL5 induces sustained insulin release in mouse islets, while in human islets, insulin release does not sustain during exposure, indicating species differences in response. Nonetheless, glucose tolerance improved in WT and insulin-resistant ob/ob mice treated with glucose solution and CCL5. However, no effect was observed in reducing insulin resistance in mice 21.

## Role of GPR75 in Metabolic Dysfunction-Related Steatosis Liver Disease (MASLD)

Akbari et al. identified 16 genes whose expression is significantly correlated with BMI. Among them, GPR75 is the gene most closely associated with decreased BMI due to functional loss 15. Through exome sequencing of hundreds of thousands of individuals from various populations including the United States, United Kingdom, and Mexico, researchers estimated rare coding variant associations with BMI, commonly used as a measure of obesity. Among genes overexpressed in the hypothalamus, protein truncation variants of GPR75 were linked to lower BMI, resulting in a weight reduction of 1.8 kg/m² and a surprising 54% lower obesity risk among heterozygous carriers 15. Further validating these findings, Akbari et al. utilized diet-induced obesity mouse models to demonstrate GPR75's effects in an allelic dosage-dependent manner using GPR75 heterozygous (GPR75^+/-^) and complete gene knockout (GPR75^-/-^) mice. Results indicated a 25% reduction in weight gain in GPR75 heterozygous mice and a 45% reduction in weight gain in complete gene knockout mice, marking the first *in vivo* evidence of GPR75's anti-obesity effects 15.

Recent research indicates that GPR75 deficiency beneficially alters energy balance to reduce fat accumulation, rather than restricting adipose tissue expansion as observed in lipodystrophy. Moreover, both male and female GPR75 knockout mice significantly prevented high-fat diet (HFD)-induced hepatic fat accumulation and exhibited reduced severity of MASLD compared to wild-type (WT) mice 6. HFD-fed mice demonstrated lower fasting glucose levels in an allelic dosage-dependent manner. Thus, GPR^+/+^ mice exhibited the highest fasting glucose levels, followed by GPR75^+/-^ and GPR75^-/-^ mice, with reports implicating GPR75 in glucose homeostasis regulation 21. Similarly, mice carrying GPR75^-/-^ alleles resisted impairments observed in glucose tolerance tests induced by HFD, although plasma insulin levels in GPR75^-/-^ mice were significantly lower than those in HFD-fed WT mice, possibly due in part to the insulin-releasing effect of previously reported CCL5 21. Nevertheless, authors did not report results compared to WT mice, which would allow readers to contextualize these values against normal conditions. Interestingly, this study associated lower weight with lower total body fat mass, lower body fat percentage, and elevated high-density lipoprotein levels, indicating a favorable lipid profile 22. These studies underscore that targeted inactivation of GPR75 in mice prevents diet-induced obesity 22-24. Furthermore, research demonstrated that CRISPR-Cas9 technology-targeted GPR75 gene knockout mice adjusted calorie intake to maintain energy balance during palatable western diet feeding, thereby preventing MASLD 6.

Subsequent studies confirmed that GPR75 deficiency promotes mechanisms leading to lower body fat and BMI. GPR75 deficiency does not reduce fat mass by limiting adipose tissue expansion itself 6. Detailed characterization of energy homeostasis in adult GPR75^-/-^ and WT mice revealed no differences in food intake, indicating that GPR75^-/-^ mice are not hypophagic. Adult GPR75 gene knockout mice did not alter the lowest metabolic rate but exhibited increased physical activity, suggesting that GPR75 gene inactivation protects against HFD-related MASLD by maintaining energy balance, thereby preventing MASLD formation 6, 24.

However, when provided with a high-fat diet, GPR75^-/-^ mice adjusted calorie intake to maintain energy balance, indicating their ability to calibrate calorie intake based on energy demands 6. Additionally, results also identified gender effects on the degree of protection against lipid accumulation, with female GPR75^-/-^ mice showing more protection than male GPR75^-/-^ mice when fed HFD, likely due to higher physical activity and energy expenditure in females. The impact of GPR75 gene knockout on adiposity was more pronounced in female mice compared to males, suggesting potential for greater effects between females and males in future research 6. A study observed high expression levels of 20-HETE in cyp4a14^-/-^ mice. In this study, two groups of mice were induced, one fed a regular diet and the other fed a high-fat diet. Mice fed a high-fat diet gained more weight. HFD group mice were given 20-SLA as a 20-HETE antagonist. Results showed that 20-SLA reduced the impact of HFD on weight gain, normalized blood glucose and insulin levels, indicating that 20-HETE is related to obesity induced by hyperlipidemia and insulin resistance and impaired insulin signaling pathways 15.

Akbari et al. discovered through exome sequencing in different populations that GPR75 gene deficiency is significantly associated with lower BMI and weight, validated in mouse models. Further research indicates that GPR75 deficiency reduces fat accumulation by altering energy balance rather than restricting adipose tissue expansion. GPR75 knockout mice exhibit lower fasting glucose levels and improved glucose homeostasis, significantly preventing HFD-induced MASLD. The study also found that female GPR75 knockout mice are more effective than males in preventing lipid accumulation under HFD, possibly due to higher physical activity and energy consumption. Ultimately, these studies demonstrate the role of GPR75 in preventing obesity and MASLD *in vivo* by maintaining energy balance.

## Summary and Future Perspectives

Despite current research revealing the potential role of GPR75 in preventing high-fat diet-induced MASLD, several limitations remain. Primarily, most studies have been conducted in mouse models, and further validation is required to ascertain their applicability to humans. Additionally, while GPR75 knockout mice demonstrate favorable metabolic traits and energy balance, the specific mechanisms remain incompletely elucidated, particularly regarding gender-specific and genetic background differences. Furthermore, existing research primarily focuses on high-fat diet-induced MASLD, with unclear implications for other types of metabolic liver diseases. CCL5 is considered a negative regulator of GPR75. Therefore, the beneficial effects of CCL5 on the nervous system may be mediated by CCL5-induced negative regulation rather than GPR75 activation. Whether such interactions exist in other tissues and organ systems requires clarification. Future studies should further explore the role of GPR75 in diverse populations, especially regarding its potential applications in human clinical trials. Moreover, investigating the interactions of GPR75 with other metabolic pathways and its mechanisms in different organs will contribute to a comprehensive understanding of its regulatory functions in metabolic diseases. Ultimately, targeted therapies based on GPR75 hold promise as novel strategies for the prevention and treatment of MASLD and other metabolic disorders.

## Author contributions

Shuo Wang contributed to the design of the paper framework and drafting of the manuscript; Shan Gao contributed to manuscript revisions; Fei Wang provided writing guidance, supervised manuscript drafting, and finalized the manuscript.

## Abbreviations

NAFLD: Non-alcoholic fatty liver disease
NASH: non-alcoholic steatohepatitis
MAFLD: metabolic dysfunction-related fatty liver disease
MASLD: metabolic dysfunction-related steatosis liver disease
GPCRs: G-protein coupled receptors
GPR75: G protein-coupled receptor 75
20-HETE: 20-hydroxyeicosatetraenoic acid
PKC-α: protein kinase C-α
RANTES: Regulated upon Activation, Normal T-cell Expressed and Secreted
CCL5: CeC chemokine ligand 5
BMI: Body Mass Inde
HFD: high-fat diet
WT: wild-type
